# Deforestation and Bovine Rabies Outbreaks in Costa Rica, 1985–2020

**DOI:** 10.3201/eid3005.230927

**Published:** 2024-05

**Authors:** Christie Jones, Amanda Vicente-Santos, Julie A. Clennon, Thomas R. Gillespie

**Affiliations:** Emory University, Atlanta, Georgia, USA

**Keywords:** rabies, Agriculture, Animals, Cattle, Chiropteran, Conservation, Lyssavirus, Zoonoses, *Desmodus rotundus*, viruses, Costa Rica

## Abstract

In Latin America, rabies virus has persisted in a cycle between *Desmodus rotundus* vampire bats and cattle, potentially enhanced by deforestation. We modeled bovine rabies virus outbreaks in Costa Rica relative to land-use indicators and found spatial-temporal relationships among rabies virus outbreaks with deforestation as a predictor.

Costa Rica has benefited from effective vaccination campaigns to eliminate canine rabies virus infections. Still, the virus has endured, spread by vampire bats (*Desmodus rotundus*) to cattle, with rare but documented transfer from bats to humans (*1*,*2*). To determine how anthropogenic disturbance affects rabies virus incidence and risk in this system, we investigated the relationship between land-use change and documented bovine rabies virus outbreaks in Costa Rica during 1985–2020.

Since 1985, the National Animal Health Service of Costa Rica (SENASA) has conducted rabies virus surveillance on domestic animals, confirming outbreaks of >1 cases by using fluorescent antibody testing (*3*). We mapped bovine rabies outbreaks during 1985–2020 reported by SENASA with neighboring land-use data by using QGIS 3.16.2 (QGIS, https://qgis.org). Ten outbreaks from the initial SENASA report (n = 119) were removed because of inaccurate location data, leaving 109 outbreaks for our study.

To evaluate outbreak probability and distribution, we used kernel density estimations with the QGIS default bandwidth to create spatial probability estimations on the basis of known outbreaks. We used a kernel function that smoothed and interpolated probabilities across the study area. We used a kernel radius of 10 km, the maximum vampire bat foraging range, limiting interpolation to the determined area (*4*). We applied a Kulldorff retrospective space-time scan with an elliptical spatial scan by using SaTScan version 9.7 (SaTScan, https://www.satscan.org) to detect the number of outbreak locations in space and time (*5*).

We applied logistic regression by using a generalized linear mixed model R-package (https://cran.r-project.org/web/packages/lme4/lme4.pdf) to evaluate the effects of land-use factors on bovine rabies virus outbreak locations compared with random control locations (n = 119). We set the district as a random effect to account for spatial effects. We set the number of control points to match the true number of SENASA-reported outbreaks. We created control locations by using the random points function in QGIS and by using the 2005 and 2017 agricultural land-use data to bind nonoutbreak samples to areas that could house cattle. We matched controls temporally to outbreaks on the basis of the proportion of outbreaks before and after 2006. 

For each outbreak and control location, we used district-level human population density and cattle population density as explanatory variables. We used the distance to and area of forest cover from each outbreak and control location within a 10-km buffer of the location. For outbreaks and control events up to 2014, we calculated forest cover by using the 2014 aerial photograph from the Atlas of Costa Rica (http://www.kyriosoft.com/atlas). For outbreaks after 2014, we used a 2018 aerial photograph from the National Territorial Information System (https://www.snitcr.go.cr). We used human population data from 2011 for all detections up to 2011. After 2011, we used a human population density estimate based on national growth trends (*6*). We used a similar approach for cattle density data based on a dataset from 2014 (*7*).

Outbreaks occurring in the northern provinces of Alajuela and Heredia clustered on the basis on their statistically significant closeness in both location and time of occurrence (6 outbreaks during 1999–2003; log likelihood ratio 7.52; p = 0.035) (Figure). The increased number of outbreaks in southern Puntarenas Province may be because of repeated emergence given the lack of space-time clustering (Figure).

**Figure:**
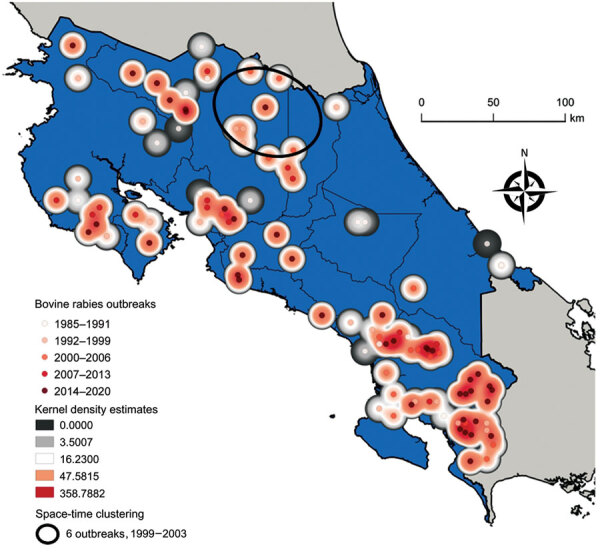
Kernel density estimations and Kulldorff space-time scan results for 109 bovine rabies outbreaks, Costa Rica, 1985–2020. Scan was limited to a 10-km distance from the epicenter of an outbreak to account for *Desmodus rotundus* vampire bat foraging ranges, enabling the detection of outbreak locations in space and time. Kernel density estimations were interpolated by using GeoDa version 1.18.0 (http://geodacenter.github.io), and the Kulldorff scan was implemented in SaTScan (https://www.satscan.org). The bovine rabies outbreak data is from the National Animal Health Service of Costa Rica. Map was created by using QGIS version 3.16.2 (https://qgis.org).

We found a positive association between the distance to forested areas and bovine rabies virus outbreaks (generalized linear mixed model estimate 4.33 × 10^−4^, SE 3.32 × 10^−1^; Z-value 1.95; p = 0.05) (Table). Each 1-km increase in distance from forested areas increased the probability of an outbreak by 4%. This finding aligns with our understanding of *D. rotundus* bat feeding preferences and rabies virus transmission risk. Decreased forested roosting site proximity appears to increase *D. rotundus* bat feeding behavior on cattle (*8*). Human and cattle densities were not associated with bovine rabies outbreaks (Table). Because human population data were unavailable until 2011 and cattle population data unavailable until 2014, the effect of those population densities may be skewed because agricultural intensification in Costa Rica has undergone major changes during the study period (*9*).

**Table:** Statistical relationship between bovine rabies virus outbreaks and relative variables of distance to forested areas, human density, and cattle density, Costa Rica, 1985–2020*

**Relative variable**	Estimate	SE	t-value	p value
**Distance to forest**	4.33 × 10^−4^	3.32 × 10^−1^	1.95	0.05†
**Human density**	–3.93 × 10^−5^	1.93 × 10^−5^	–1.53	0.13
**Cattle density**	–3.76 × 10^−5^	3.23 × 10^−5^	–0.93	0.35

*Results of a generalized linear mixed model regression analysis used to determine a statistical relationship between deforestation, human and bovine density, and bovine rabies outbreaks using data from the National Animal Health Service of Costa Rica, the 2014 Atlas of Costa Rica aerial photograph, the 2018 National Territorial Information System aerial photograph, population data, and population density estimates based on growth trends. **†p-value <0.05 is considered statistically significant. **

Our results show an association between deforestation and bovine rabies virus outbreaks, highlighting the importance of considering negative health effects in risk assessments for forest conversion proposals (*10*). Our results indicated the southern region of Costa Rica has the highest probability of bovine rabies outbreaks, indicating the need for localized, preventative interventions in the south. On the basis of recent findings, we must caution against bat culling as a response to this threat, because disrupting bat dispersal in unexpected ways may increase the spread of the rabies virus (*2*). Because rabies virus remains endemic in Latin America, an increased focus on integrating spatial, dietary, and surveillance data for *D. rotundus* bats is needed to provide additional insights into land-use effects on the persistence and spread of the rabies virus.
